# Genome-Wide Association Study Reveals the Genetic Basis of Stalk Cell Wall Components in Maize

**DOI:** 10.1371/journal.pone.0158906

**Published:** 2016-08-01

**Authors:** Kun Li, Hongwu Wang, Xiaojiao Hu, Zhifang Liu, Yujin Wu, Changling Huang

**Affiliations:** Institute of Crop Science, Chinese Academy of Agricultural Sciences, Beijing, 100081, China; Iowa State University, UNITED STATES

## Abstract

Lignin, cellulose and hemicellulose are the three main components of the plant cell wall and can impact stalk quality by affecting cell wall structure and strength. In this study, we evaluated the lignin (LIG), cellulose (CEL) and hemicellulose (HC) contents in maize using an association mapping panel that included 368 inbred lines in seven environments. A genome-wide association study using approximately 0.56 million SNPs with a minor allele frequency of 0.05 identified 22, 18 and 24 loci significantly associated with LIG, CEL and HC at P < 1.0×10^−4^, respectively. The allelic variation of each significant association contributed 4 to 7% of the phenotypic variation. Candidate genes identified by GWAS mainly encode enzymes involved in cell wall metabolism, transcription factors, protein kinase and protein related to other biological processes. Among the association signals, six candidate genes had pleiotropic effects on lignin and cellulose content. These results provide valuable information for better understanding the genetic basis of stalk cell wall components in maize.

## Introduction

Maize (*Zea mays L*.) is one of the three most important staple crops, providing protein, lipids and vitamins for billions of people around the world. It, along with silage maize, also serves as an important energy resource for ruminant animal. Although the energy value of forage plants is lower and more variable[1], their stover is highly useful in animal husbandry. Thus, improving the feeding value of forage crops is key target of silage maize breeding.

Plant cell walls make a large contribution to forage utilization [2], whereas, the limited digestion of fiber in the rumen makes the feeding value of forage lower than grain [1, 3]. The cell wall of maize plants consists mainly of cellulose, hemicellulose and lignin. The fiber and lignin content, is negatively correlated with cell wall digestibility[4, 5]. On the other hand, plant cell wall components are also related with resistance to lodging, pest (such as corn border), disease and abiotic stress [6–10]. In breeding programs, selecting for high stalk strength and resistance to corn border causes an increase in the cell wall components [11, 12]. Selection for cell wall components can also affect stover digestibility [13]. To understand the genetic correlation between these traits, several linkage analysis studies were performed to detect quantitative trait loci (QTL) for cell wall components, digestibility traits and stalk strength [14–28]. Underlying these QTL, a number of candidate genes were found to be involved in cellulose and lignin biosynthesis, which can help us better understand genetic architecture of cell wall components.

QTL mapping in bi-parent populations identified many genomic regions related to cell wall components, although the low density of the linkage maps and few recombination events in the mapping population limited the mapping resolution and led to a large confidence interval for each QTL. In recent years, genome-wide association studies become a powerful tool for dissecting the genetic basis of complex traits and identifying favorable alleles or haplotypes for target traits [29]. Compared with linkage analysis, association mapping requires less time to develop a mapping population [30], and evaluates more alleles in a diverse population simultaneously [31]. Additionally, based on linkage disequilibrium in natural populations, association mapping uses abundant historical recombination to improve the resolution of the identified QTL. In maize, the LD (linkage disequilibrium) decays rapidly [29], and association analysis with high density markers covering the whole genome can accurately narrow down the association confidence interval into a small genomic region even to the gene level. Up until now, GWAS have been widely used on maize to identify significant associations related to grain quality traits [32–36], agronomic traits [37–39], yield traits [40], disease resistance [41–47] and stress tolerance [48–51]. Nevertheless, as far as we are concerned, no GWAS was undertaken to detect the associations related with cell wall components in maize. Whereas, GWAS for cell wall related traits were performed in other plants and obtained many potential candidate genes. Associations for cellulose and (1,3;1,4)-β-glucan content were scanned in two barley association panels, and identified a set of CELLULOSE SYNTHASE-LIKE (Csl) genes and genes co-expressed with the CELLULOSE SYNTHASE A gene family [52, 53]. In *Populus*, a GWAS study was conducted to scan 29,233 high quality SNPs in a population of 334 *Populus trichocarpa* individuals, and found 141 significant SNPs associate with 16 wood characteristics traits [54]. These results provide unique insight into the genetic basis of cell wall related traits.

In this study, we performed GWAS on a set of 368 inbred lines to analyze cell wall components. The objective s of this study were (1) to evaluate variations in stalk cell wall components in the maize natural population; (2) to identify significant SNPs or loci related to the cell wall components and (3) to dissect the genetic basis of cell wall component traits in maize stalks.

## Materials and Methods

### Plant materials and filed experiments

The association mapping panel consisted of 368 diverse inbred lines (AM368), including resources from the International Maize and Wheat Improvement Center (CIMMTY), China and the USA. The lines from CIMMTY consisted mainly of tropical or sub-tropical germplasm. Detailed information about AM368 was described in a previous study [34]. These inbred lines were planted in Hainan, Yunnan in 2010; in Hainan, Henan and Yunnan in 2011; and in Hainan and Yunnan in 2012. A randomized block design was constructed at all locations without replication. Each line was planted in a single row (2.5 m in length) of 11 plants at a density of 60,000 plants/ha. Adjacent rows were spaced 0.67 m apart.

### Phenotype evaluation

After harvest, the second to fifth internodes above the ground of six plants from each inbred line were collected. All samples were immediately enzyme-deactivated at 105°C for 30 min in a forced air oven and air-dried for 10–14 days. Dried stalk samples were ground with a mill and sieved with the 0.1mm mesh. LIG, CEL and HC were detected by near-infrared reflectance spectroscopy (NIRS). Before measuring, stalk samples were dried at 45°C for 48 h to exclude any moisture. Samples were scanned through a near-infrared reflectance spectrophotometer (VECTOR22/N; BURKER Optik, Ettlingen, Germany). The amounts of LIG, CEL and HC were determined using NIRS prediction equations developed for maize plant. The content of each of these three components in maize was expressed in percent of dry matter. Modified partial least squares implemented in OPUS 6.0 Bruker software was used for fitting calibration equations [55]. The coefficients of determination for cross-validation (*R*^*2*^_*CV*_) and external validation (*R*^*2*^_*Val*_) were 90.5% and 92.7% for LIG, 94.0% and 96.7% for CEL, and 89.7% and 91.2% for HC.

### Genotyping

Genotyping of the association mapping panel consisted of two sets, including the MaizeSNP50 BeadChip containing 56,110 SNPs and 1.03 million high quality SNPs detected by RNA sequencing[34, 56]. By combining these two sets of genotypes and removing the duplicate SNPs, a total of 559,285 high quality SNPs with minor allele frequencies (MAF) larger than 0.05 were used in this study.

### Phenotypic data analyses

The GLM procedure in SAS9.3 (SAS Institute) was performed to dissect the variance of phenotypes in different environments. The model used for analysis of variance was: y = *μ* + *e*_*l*_ + *f*_*i*_ + *ε*_*li*_, where *μ* is the grand mean of the target trait, *e*_*l*_ is the environmental effect of the “l”th environment, *f*_*i*_ represents the genetic effect of the “i”th line, and *ε*_*li*_ is denoted as the residual error. The broad-sense heritability was calculated as $h^{2}=\sigma_{g}^{2}/\left(\;\sigma_{g}^{2}+\sigma_{\varepsilon }^{2}/e\right)$, where $\sigma_{g}^{2}$ represents the genetic variance, $\sigma_{\varepsilon }^{2}$ is the residual error variance item, *e* is the number of environments. A 95% confidence interval of *h*^2^ was calculated following the method by Knapp et al. [57].

To eliminate the effect of environment variation, we fitted a mixed linear model to calculate the best linear unbiased prediction (BLUP) values for each trait in each line:*y*_*i*_ = *μ* + *g*_*l*_ + *e*_*i*_ + *ε*_*i*_. In this equation, *y*_*i*_ represents the phenotype of the “i”th line, *μ* is the grand mean value of the target trait in all environments, *g*_*i*_ is denoted as genetic effect, *e*_*i*_ is the environmental effect, and ε_*i*_ is the random error. BLUP estimation was obtained by using the MIXED procedure (PROC MIXED) in SAS9.3 (SAS Institute), which should be denoted as the sum of the grand mean and the genetic effect of each line. The BLUP values of each line were used as phenotype values for association mapping.

### Genome-wide association analysis and gene annotation

GWAS were performed on cell wall component traits by fitting population structure (Q) and relative kinship (K) in a mixed linear model (MLM), which was implemented in TASSEL 4.1.26 [58]. Population structure and kinship matrices were estimated in a previous study [34]. With the Bonferroni correction threshold for GWAS, we found only one SNP (chr2.S_1360791) associated to HC and none significant association for CEL and LIG. Therefore, a less strict criterion of P < 1×10^−4^ was chosen to determine significant SNPs for the three traits. All candidate genes were annotated according to the information available in MaizeSequence (http://ensembl.gramene.org/Zea_mays/Info/Index) and the MaizeGDB database (http://www.maizegdb.org/gbrowse).

## Results

### Phenotypic variation

**Fig 1:**
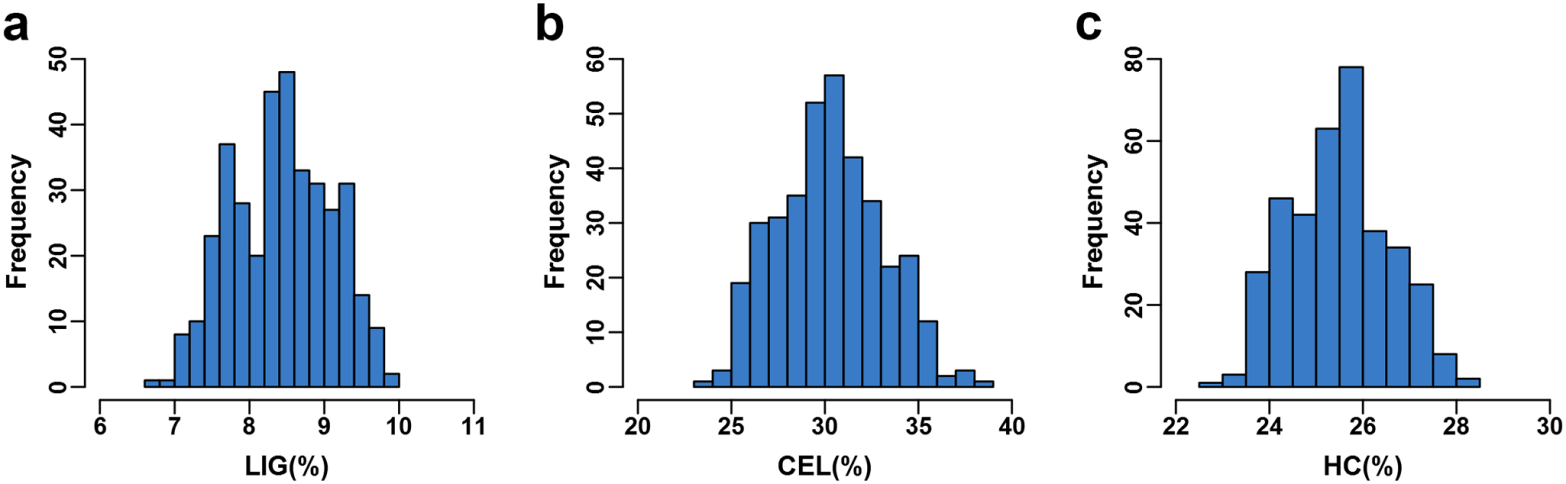
Frequency distributions of LIG, CEL and HC in a maize association mapping panel.

With the association mapping population including 368 diverse inbred lines, the extent of the phenotypic variations was estimated for all three cell wall components, LIG, CEL and HC. These traits showed significant diversity with a normal distribution (Fig 1), with mean values of 8.43±0.67% (LIG), 30.26±2.81% (CEL) and 25.47±1.08% (HC) (Table 1). The correlation among each trait revealed that HC had a weak correlation with both LIG and CEL (r = 0.24 and 0.26, P < 0.01), while CEL was positively correlated with LIG (r = 0.85, P < 0.01). Based on phenotypic values measured in seven environments, the analysis of variance revealed significant differences caused by the genotype effect (P < 0.01) for each trait (Table 1). We observed that all the three traits possess moderate to high heritability (ranging from 0.68–0.83), suggesting that variations of cell wall components are affected mainly by genetic factors.

**Table 1:** Phenotypic variation, heritability and correlation.

Traits	Descriptive Statistics			Mean Squares		H^2^^b^	Confidence Interval ^c^	Correlation		
	Min	Max	Mean±Sd	Env^a^	Geno^a^			LIG	CEL	HC
LIG(%)	6.78	9.95	8.43±0.67	179.98**	3.62**	0.83	0.81–0.85	1		
CEL(%)	23.55	38.24	30.26±2.81	7990.41**	70.56**	0.79	0.76–0.82	0.84**	1	
HC(%)	22.74	28.26	25.47±1.08	4736.96**	14.01**	0.68	0.63–0.72	0.23**	0.22**	1

**Significant at P < 0.01

^a^ Mean square values for environmental and genotypic factors.

^b^ Broad sense heritability.

^c^ 95% confidence interval of broad sense heritability.

### Genome wide association mapping

**Fig 2:**
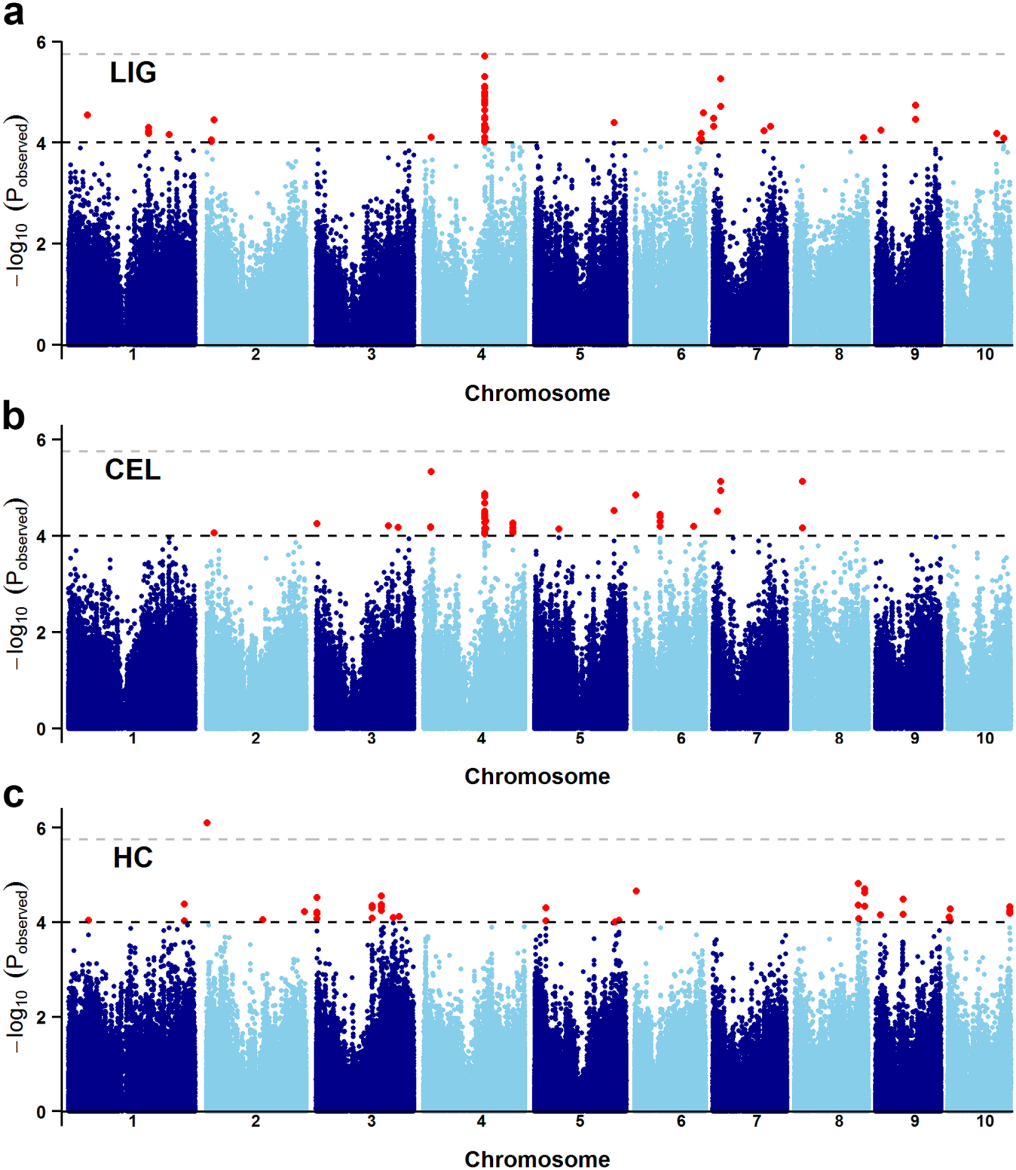
Manhattan plots of GWAS for cell wall components in a maize association panel. Manhattan plots for LIG, CEL and HC are shown in **a**, **b** and **c**, respectively. Grey and black dashed lines correspond to the thresholds of Bonferroni correction (P < 1.8×10^−6^) and P < 1×10^−4^, and red dots represent the significant SNPs for target traits (P < 1×10^−4^).

To dissect the genetic basis of natural variations in three cell wall components, we performed a GWAS study by fitting a mixed linear model with population structure and familial relatedness. As shown in Manhattan plots (Fig 2), the Bonferroni correction of P < 1.8×10^−6^ (1/N, N represents the number of markers used in GWAS) was too strict for detecting associations related to the three traits, and therefore we chose P < 1×10^−4^ as the threshold for the present study. The Q-Q plot (S2 Fig) for GWAS based on BLUP value of each trait revealed that the associations were well controlled for population structure. 22 unique loci were identified associated with LIG, which were distributed on all chromosomes except chromosome 3 (Fig 2a, Table 2). 64 SNPs covering 18 loci were found significant for CEL, and 1/3 of these loci were located on chromosome 4 (Fig 2b, Table 2). 50 SNPs were significantly associated with HC (Fig 2c), which contains 24 unique loci located on all chromosomes except 4 and 7. The phenotypic variation denoted by each locus of each trait ranged from 4%-7%.

**Table 2:** SNPs and candidate genes significantly associated with LIG, CEL and HC.

Trait	SNP^a^	Chr	Position^b^	Alleles^c^	MAF^d^	P value	(R^2^)^e^	Gene	Annotation
LIG	chr1.S_45949700	1	45949700	C/A	0.07	2.83E-05	0.05	GRMZM2G007206	Tetraspanin family protein
LIG	chr1.S_191089782	1	191089782	G/A	0.14	4.93E-05	0.05	GRMZM2G005652	Ypt/Rab-GAP domain of gyp1p superfamily protein
LIG	chr1.S_239615661	1	239615661	C/A	0.08	6.84E-05	0.04	GRMZM2G010871	HSF-transcription factor
LIG	chr2.S_11830421	2	11830421	T/C	0.19	8.66E-05	0.04	GRMZM2G392125	Xyloglucan endotransglucosylase/hydrolase protein
LIG	chr2.S_12257289	2	12257289	A/G	0.28	9.48E-05	0.04	GRMZM2G374203	Disease resistance/zinc finger/chromosome condensation-like region protein
LIG	chr2.S_18351185	2	18351185	A/G	0.18	3.48E-05	0.05	GRMZM2G036996	Unknown function
LIG	chr4.S_17501001	4	17501001	T/C	0.12	7.71E-05	0.04	GRMZM2G106798	SBP transcription factor
LIG	chr4.S_145921053	4	145921053	C/T	0.39	1.93E-06	0.06	GRMZM2G133444	Unknown function
LIG	PZE-104075102	4	147587451	G/A	0.47	5.12E-05	0.05	GRMZM2G148355	NAD(P)-binding Rossmann-fold superfamily protein
LIG	chr5.S_188832912	5	188832912	T/G	0.20	3.99E-05	0.05	GRMZM2G169994	RING membrane-anchor 1
LIG	chr6.S_155653406	6	155653406	T/C	0.46	8.47E-05	0.04	GRMZM2G140817	ZmC3H2 (coumarate-3- hydroxylase)
LIG	chr6.S_159383339	6	159383339	C/T	0.33	6.57E-05	0.04	GRMZM2G331833	CLP protease regulatory subunit X
LIG	chr6.S_164498311	6	164498311	C/T	0.14	2.53E-05	0.05	GRMZM2G031200	AtSND2/SND3-like NAC type transcription factor
LIG	chr7.S_2739958	7	2739958	T/G	0.14	3.28E-05	0.05	Intergenic	
LIG	chr7.S_19347352	7	19347352	G/C	0.12	5.41E-06	0.06	GRMZM2G042627	Kinase associated protein phosphatase
LIG	chr7.S_122072420	7	122072420	C/T	0.17	5.74E-05	0.05	GRMZM2G086714	Unknown function
LIG	chr7.S_137488120	7	137488120	G/C	0.08	4.75E-05	0.05	GRMZM2G144275	bHLH-transcription factor
LIG	PZA00516.2	8	164866552	G/A	0.32	7.99E-05	0.04	GRMZM2G050803	Unknown function
LIG	chr9.S_13460855	9	13460855	A/G	0.06	5.62E-05	0.05	GRMZM2G409974	ARR-B-transcription factor
LIG	chr9.S_96077879	9	96077879	T/A	0.43	1.80E-05	0.05	GRMZM2G704277	Calcium-binding protein
LIG	chr10.S_117571849	10	117571849	G/C	0.35	6.49E-05	0.04	GRMZM2G072322	Late embryogenesis abundant domain-containing protein
LIG	chr10.S_134054013	10	134054013	T/C	0.33	8.08E-05	0.04	GRMZM2G427097	Glutamate dehydrogenase 2 (GDH2)
CEL	chr2.S_18351185	2	18351185	A/G	0.18	8.69E-05	0.04	GRMZM2G036996	Unknown function
CEL	chr3.S_1691708	3	1691708	C/A	0.16	5.50E-05	0.05	GRMZM2G308619	Protein kinase
CEL	chr3.S_172376137	3	172376137	A/G	0.43	6.13E-05	0.04	GRMZM2G167253	LRR-type receptor protein kinase
CEL	SYN33683	3	195268058	G/A	0.42	6.71E-05	0.05	GRMZM2G163407	CRR7- novel subunit NDH complex
CEL	chr4.S_17095498	4	17095498	A/G	0.18	6.44E-05	0.04	GRMZM2G045987	COPII
CEL	chr4.S_17501001	4	17501001	T/C	0.12	4.65E-06	0.06	GRMZM2G106798	SBP transcription factor
CEL	chr4.S_145920807	4	145920807	A/C	0.47	1.34E-05	0.05	GRMZM2G133444	Unknown function
CEL	PZE-104075102	4	147587451	G/A	0.47	4.92E-05	0.05	GRMZM2G148355	NAD(P)-binding Rossmann-fold superfamily protein
CEL	PZE-104075114	4	147652919	A/G	0.45	7.02E-05	0.05	GRMZM2G134752	LIM-transcription factor 8
CEL	chr4.S_212042669	4	212042669	A/G	0.49	5.46E-05	0.05	GRMZM2G702806	2-oxoglutarate (2OG) and Fe(II)-dependent oxygenase superfamily
CEL	chr5.S_57247506	5	57247506	A/G	0.31	7.14E-05	0.04	GRMZM2G053066	RHOMBOID-like protein 14
CEL	chr5.S_188831963	5	188831963	T/C	0.15	3.03E-05	0.05	GRMZM2G169994	RING membrane-anchor 1
CEL	chr6.S_2589885	6	2589885	G/C	0.37	1.41E-05	0.05	GRMZM2G116685	CRINKLY4-like receptor protein kinase family protein
CEL	SYN21998	6	60973648	A/G	0.42	3.59E-05	0.05	GRMZM2G456023	START domain containing protein
CEL	chr6.S_141413297	6	141413297	G/C	0.38	6.41E-05	0.04	GRMZM2G012724	WRKY-transcription factor
CEL	chr7.S_11665336	7	11665336	G/C	0.28	3.10E-05	0.05	AC148167.6_FG001	TPX2 (targeting protein for Xklp2) protein family
CEL	chr7.S_19347352	7	19347352	G/C	0.12	7.50E-06	0.06	GRMZM2G042627	Kinase associated protein phosphatase
CEL	chr8.S_18967150	8	18967150	G/C	0.10	7.33E-06	0.06	GRMZM2G047949	RNA helicase, ATP-dependent, SK12/DOB1 protein
HC	chr1.S_47995277	1	47995277	T/C	0.17	9.04E-05	0.04	GRMZM2G017400	Unknown function
HC	chr1.S_276323701	1	276323701	C/T	0.36	4.12E-05	0.05	GRMZM2G017186	Cell wall.degradation.cellulases and beta -1,4-glucanases
HC	chr2.S_1360791	2	1360791	C/A	0.14	7.85E-07	0.07	GRMZM2G457621	Rubisco Accumulation Factor 1
HC	chr2.S_134608512	2	134608512	C/T	0.05	8.69E-05	0.04	GRMZM2G174919	Cleavage stimulating factor 64 (CSTF64)
HC	chr2.S_232987292	2	232987292	C/G	0.24	5.93E-05	0.04	GRMZM2G082809	TPX2 (targeting protein for Xklp2) protein family
HC	chr3.S_2072018	3	2072018	T/G	0.45	2.98E-05	0.05	GRMZM2G153138	Thioesterase superfamily protein
HC	chr3.S_133787782	3	133787782	A/C	0.41	4.93E-05	0.04	GRMZM2G155974	(GSH2, GSHB) glutathione synthetase 2
HC	chr3.S_133889119	3	133889119	A/C	0.41	4.49E-05	0.05	GRMZM2G116204	Auxin-binding protein 1
HC	chr3.S_155858370	3	155858370	G/A	0.25	2.76E-05	0.05	GRMZM2G026758	NAD(P)-binding Rossmann-fold superfamily protein
HC	chr3.S_183639338	3	183639338	C/T	0.26	7.92E-05	0.04	GRMZM2G432662	Prenylated RAB acceptor 1.B4
HC	PZE-103142610	3	198039525	A/G	0.35	7.54E-05	0.04	Intergenic	
HC	chr5.S_27102301	5	27102301	G/A	0.09	4.88E-05	0.04	GRMZM2G174145	Pyridoxal phosphate (PLP)-dependent transferases superfamily protein
HC	chr5.S_191774922	5	191774922	C/G	0.46	9.75E-05	0.04	GRMZM2G118731	Chaperone protein dnaJ putative expressed
HC	chr5.S_200651833	5	200651833	T/C	0.15	9.09E-05	0.04	GRMZM2G179147	abh1—abscisic acid 8'-hydroxylase1
HC	chr6.S_3974370	6	3974370	G/C	0.28	2.16E-05	0.05	GRMZM2G175995	Unknown function
HC	chr8.S_152112653	8	152112653	T/C	0.48	4.37E-05	0.05	GRMZM2G164640	Signaling.receptor kinases
HC	chr8.S_152130649	8	152130649	C/G	0.07	1.53E-05	0.05	GRMZM2G393150	LTPL150—Protease inhibitor/seed storage/LTP family protein precursor expressed
HC	chr8.S_152593975	8	152593975	A/G	0.10	8.38E-05	0.04	GRMZM2G060886	S-adenosyl-L-methionine-dependent methyltransferases
HC	chr8.S_166787472	8	166787472	C/G	0.23	1.94E-05	0.05	Intergenic	
HC	chr9.S_11457267	9	11457267	G/T	0.35	6.98E-05	0.04	Intergenic	
HC	chr9.S_65522135	9	65522135	T/G	0.50	3.25E-05	0.05	GRMZM2G343048	Unknown function
HC	chr10.S_4094497	10	4094497	T/A	0.08	7.64E-05	0.04	Intergenic	
HC	chr10.S_6373492	10	6373492	T/A	0.15	5.16E-05	0.04	GRMZM2G148404	Unknown function
HC	chr10.S_147932122	10	147932122	C/T	0.07	4.65E-05	0.05	GRMZM2G069389	Unknown function

^a^ Leading SNP of each significant loci associated with each trait.

^b^ Physical position of the leading SNP according to version 5b.60 of the maize reference sequence (http://ensembl.gramene.org/Zea_mays/Info/Index).

^c^ The allele before the slash represents the favorable allele.

^d^ MAF, minor allele frequency.

^e^ R^2^, proportion of the phenotypic variance explained by the SNP.

### Candidate genes co-localized with associated SNPs

Based on the available B73 reference genome information, candidate genes containing leading SNPs for each trait were identified. Excluding 5 of 64 leading SNPs located within inter-genic region, 21, 18 and 20 genes were found to be associated with LIG, CEL and HC, respectively (Table 2), and 6 genes were associated with both CEL and LIG. Beside several genes encoding for uncharacterized proteins, the majority of the predicted candidate genes encoded transcription factors, protein kinases, and enzymes involved in cell wall metabolism. The rest of candidate genes encode proteins with specific domains that could not be directly correlated with cell wall components.

**Fig 3:**
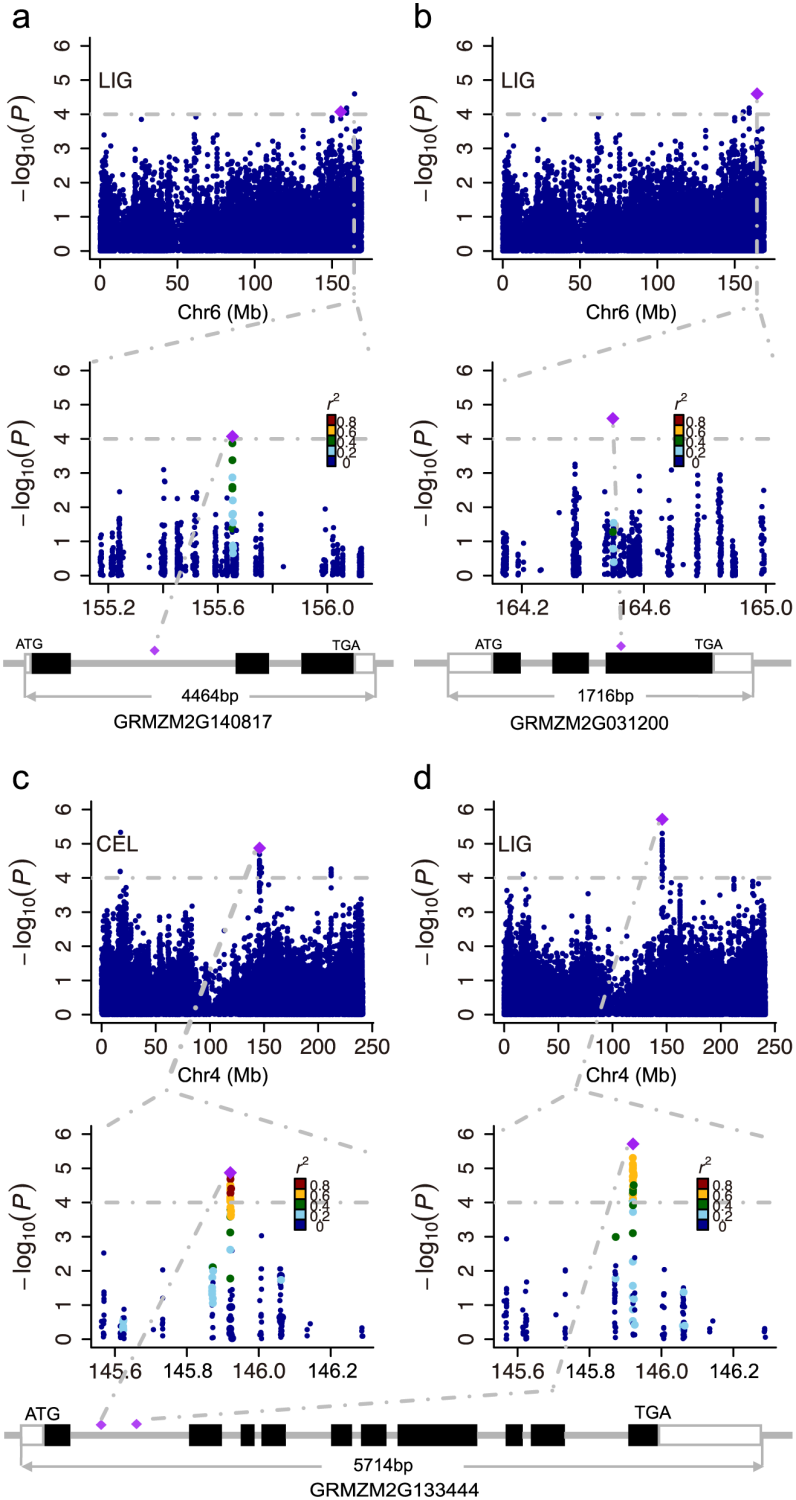
Association and genomic location of known and new loci associated with LIG and CEL. **(a-d)** *GRMZM2G140817* (*ZmC3H2*) **(a)** and *GRMZM2G031200* **(b)** are associated with LIG. *GRMZM2G133444* is associated with CEL **(c)** and LIG **(d)** with different leading SNPs. (Top) Association results of cell wall components for one chromosome. (Middle) A 0.5-Mb region on each side of the leading SNP (the SNP with the lowest P value), which is denoted by a purple diamond. The position of the leading SNP is indicated with grey dashed line. The color of the remaining SNPs reflects r^2^ values with the most significantly associated SNP. Dashed horizontal lines depict the significance threshold (1×10^−4^). (Bottom) Gene structure according to the information from the B73 genome sequence in the GRAMENE database (http://ensembl.gramene.org/Zea_mays/Info/Index).

Remarkably, we found a set of genes involved in the cell wall component biosynthesis pathway or directly related to secondary cell wall modifications. The SNP on chromosome 6 (chr6.S_155653406) significantly associated with LIG was found located within the gene model *GRMZM2G140817* (Fig 3a), which encodes a coumarate 3-hydroxylase (C3H) and plays a vital role in the lignin biosynthesis pathway [59]. This protein is one of three cytochrome P450 enzymes which catalyze hydroxylation reactions in the lignin pathway. Another significant SNP located on chromosome 6 at position 164,498,311 is contained in the gene region of *GRMZM2G031200* that encodes a secondary wall-associated NAC domain protein (Fig 3b), which is a transcription factor involved in the regulation of secondary cell wall biosynthesis [60]. The most associated SNP, chr6.S_164498311, has a C/T variant and results in an arginine (R) to tryptophan (W) amino acid change on the third exon of *GRMZM2G031200*. In addition, the most significant association signal for LIG was identified on chromosome 4 at position 145,921,053 (Chr4_145921053) (Fig 3d). Due to the linkage disequilibrium, the adjacent 42 continuous SNPs of the leading SNP showed a strong association with LIG (Fig 3d). These SNPs were located within gene model *GRMZM2G133444*, which encodes an uncharacterized protein that has not been correlated with the cell wall component biosynthesis pathway. Interestingly, this candidate gene was also found associated with CEL (Fig 3c), but with a different leading SNP from Chr4_145921053.

## Discussion

### Phenotypic variation and heritability

Cellulose, hemicellulose and lignin are three organic compounds of the plant cell walls. These aromatic polymers and polysaccharides bond together and provide the basic skeleton for the secondary cell wall. Lignin content, structure and cross-linking between cell wall components has a significant impact on cell wall digestibility [1]. Several studies about the association between lignin pathway genes and cell wall digestibility were performed in association mapping populations with sample size of less than 50 [61–64]. In this study, 1.2–1.6 fold variations of cell wall components were detected in a larger sample size of association mapping panel which consisted of 368 inbred lines across whole world. According to the information of population structure in previous study [65], phenotypic variation of LIG and CEL between sub-groups was compared and obvious differences were identified (S1 Fig). LIG and CEL levels in the stiff stalk (SS) sub-group was higher than those in the non-Stiff stalk (NSS) and mixed group (Mixed) significantly (p < 0.05), and relatively higher than the Tropical and Sub-tropical group (TST), although it was not statistically significant. These results are not surprising given that SS group lines experienced stalk quality selection which may have caused the increase of the cell wall components.

The analysis of variance revealed that both environment and genetic effects contributed significantly to the phenotypic variation. To avoid the influence of the environment, a mixed linear model was fitted to calculate the BLUP value of each trait. The heritablities of the cell wall components in this study ranged from 0.68 to 0.83,which were as well as in the previous linkage studies that ranged from 0.51 to 0.92 [17, 20, 66, 67].These results indicate that these traits are suitable for dissecting the genetic architecture using GWAS.

### Genetic architecture of cell wall components

In previous linkage studies, numerous QTL for the cell wall components and the digestibility trait were detected in diverse populations [14–27]. These QTL were reviewed and projected on a consensus map using meta-analysis [68]. All of the identified cell wall components-related QTL covered 77% of the maize genome, and 42 meta-QTL for cell wall components were identified using meta-analysis that contained 26% of the maize genome. These results suggest that the genetic basis for the cell wall components is complicated and controlled by a large number of genome regions. In this study, we performed GWAS on three cell wall components with more than 550, 000 SNPs across the entire genome. Population structure and kinship matrices were accounted to reduce the spurious association. According to the Quantile-Quantile plot of the association analysis (S2 Fig), false positive associations were well controlled with the Q+K model. However, the Bonferroni correction threshold (P = 1/N, N represents the number of markers used in GWAS) was too stringent for the present study. A less stringent cutoff of 1×10^−4^ was applied for significant association detection. And we found 82, 64 and 50 associated SNPs, covering 22, 18 and 24 unique loci, that were significantly associated with LIG, CEL and HC, respectively. Each SNP can explain a small portion of the phenotypic variation, which also reveals that cell wall components are controlled by numerous minor-effect genes or QTL.

### Candidate genes for cell wall components

Based on the association signals associated with the target traits, we found a number of candidate genes for the cell wall components. A SNP (chr6.S_155653406) associated with LIG was located within the first intron of *GRMZM2G140817* and didn’t caused any change in alternative splicing. *GRMZM2G140817* encodes coumarate 3-hydroxylase (*C3H*), a cytochrome P450 dependent monooxygenase. This enzyme catalyzes the hydroxylation reaction of the aromatic ring in guaiacyl (G) and syringyl (S) monolignol synthesis [59]. In *Arabidopsis*, *reduced epidermal fluorescence* (*ref8*) mutant deficient in *C3H* showed a reduction in lignin content and increased accumulation of p-hydroxyphenyl (H) monolignol [69]. Downregulation of *C3H* in alfalfa plants also caused a severe decrease in total lignin and an increase in the H monomer, which demonstrated that low *C3H* activity is correlated with the inhibitory effect of transformation from p-coumaroyl CoA to caffeoyl shikimate [70]. Recently, a *ZmC3H1* knock-down study revealed a moderate increase in H monomers and cell wall degradation changes, along with expression reduction of *ZmC3H1*. The author concluded that the moderate effect of *ZmC3H1*, when compared to the corresponding *C3H* in Arabidopsis and alfalfa, was caused by the compensation effect of *ZmC3H2* [71]. All these evidence described above suggest that *ZmC3H2* may be a candidate gene related with lignin content as we found in present study. In addition, another candidate gene, *GRMZM2G031200*, which co-localized with a significant SNP (chr6.S_164498311) associated with LIG, was also found located on chromosome 6. This gene encodes an SND2/SND3-like transcription factor that belongs to the secondary wall-associated NAC domain protein (SND) family, and SND2 and SND3 have been shown to induce secondary wall biosynthesis genes in *Arabidopsis* [60]. Furthermore, *GRMZM2G140817* and *GRMZM2G031200* were identified localized within the QTL interval in bin 6.06 which was identified related with lignin content in F288 ×F271 RIL population[27, 72]. These results provide more supportive evidence that these two candidate genes influence lignin content.

Among the candidate genes associated with CEL, none was specifically involved in the cellulose biosynthesis pathway. On chromosome 7, a candidate gene (*GRMZM2G042627*) associated with CEL that encodes a kinase associated protein phosphatase, which was also shown to be related to resistance to the Mediterranean corn borer (MCB, *Sesamia nonagrioides L*.) in another maize association panel [51]. Considering the impact of cell wall composition on maize resistance to pests (including Mediterranean corn borer) [8], co-localization of associations related to cellulose content and MCB may be the result of the pleiotropy of this stress response regulation gene.

The candidate gene (*GRMZM2G017186*) which encodes a beta-glucosidase and is located on chromosome 1, was found to be associated with HC. This enzyme is involved in cell wall degradation and catalyzes the degradation reaction of cellulose. *In vitro*, cellulase mixed with beta-glucosidase has been demonstrated having cross activity of xylanase and *β*-xylosidase [73]. Since no evidence exists in terms of the overlapping activity of cellulase and hemicellulase *in vivo*, the association between *GRMZM2G017186* and hemicellulose content needs to be validated with further studies.

The association between maize cell wall digestibility and candidate gene polymorphisms was reported for ten key enzyme genes involved in lignin biosynthesis [61–64]. Several polymorphisms on the *PAL* gene were associated with neutral detergent fiber and *in vitro* digestibility of organic matter (IVDOM); however, the results of the association analysis were influenced by population structure [63]. *ZmC3H1* and *F5H* were also found to be associated with forage quality traits, while the associations of these two genes were not significant in multiple tests [64]. Among the remaining genes, only the genetic variations in *4CL1*, *CCoAOMT2* and *ZmPox3* were found to be significantly associated with cell wall related traits [61, 62, 64]. In the present study, among the association candidate genes other than *ZmC3H2* identified by GWAS, none was found involved in the lignin biosynthesis process. Taking previous results into account, it appears that the lignin content is mainly controlled by functional variations in several genes which play key roles in the metabolic pathway.

### Co-localization of associations for the different cell wall components

In addition to the candidate genes mentioned above, we found six common associated genes for cellulose and lignin. These genes were annotated encoding a kinase associated protein phosphatase (*GRMZM2G042627*), an SBP transcription factor (*GRMZM2G106798*), an NAD(P)-binding Rossmann-fold superfamily protein (*GRMZM2G148355*), a RING membrane-anchor protein (*GRMZM2G169994*) and two uncharacterized proteins (*GRMZM2G036996* and *GRMZM2G133444*). The kinase-associated protein phosphatase gene, *GRMZM2G042627*, which has also been found co-localized with MCB related associations [51], was reported to regulate responses to biotic stress[74]. Other genes didn’t have direct correlation with cell wall components. In light of the strong correlation between CEL and LIG detected in this study (Table 1) and no obvious intersection between two metabolic pathways, the co-localization of the association signals needs further evaluation.

## Conclusions

In the present study, we dissected the genetic architecture of cell wall components using GWAS, and found that cell wall components are controlled by many minor-effect genes. Candidate genes for each component were annotated, most of which encode transcription factors, protein kinases, enzymes involved in cell wall biosynthesis and proteins involved in other biology process. Underlying the significant associations, we found several potential candidate genes with some evidence support, which included a *C3H* gene involved in lignin pathway and a NAC domain transcription factor related with secondary cell wall modification. With these results, we now have better understanding of the genetic basis of the composition of stalk’s cell wall, and can improve these traits through genome-wide selection. However, additional validation, such as candidate gene–based association analysis, fragment introgression and transgenic methods, is necessary to verify these associations.

## Supporting Information

S1 Fig Phenotypic distribution of three cell wall components in different sub-populations. Boxplot for LIG, CEL and HC in each group are shown in **a**, **b** and **c**, respectively. TST, tropical and sub-tropical group; SS, stiff stalk group; NSS, non-stiff stalk group; MIXED, mixed group.
(TIFF)

S2 Fig Quantile-Quantile (QQ) plots for cell wall components in a maize association panel. QQ plots for LIG, CEL and HC are shown in **a**, **b** and **c**, respectively. Horizontal grey solid line and black dashed line correspond to the thresholds of Bonferroni correction and 1×10^−4^.
(TIFF)

S1 Table The phenotypic evaluation of cell wall components in association panel across environments. The phenotypic data are the BLUP values across multiple environments.
(XLSX)
