# Supplementary figures and images for: Genome-Wide Association Study Reveals the Genetic Basis of Stalk Cell Wall Components in Maize
Source: PLoS One. 2016 Aug 1;11(8):e0158906. doi: 10.1371/journal.pone.0158906 (PMC4968789; doi:10.1371/journal.pone.0158906)

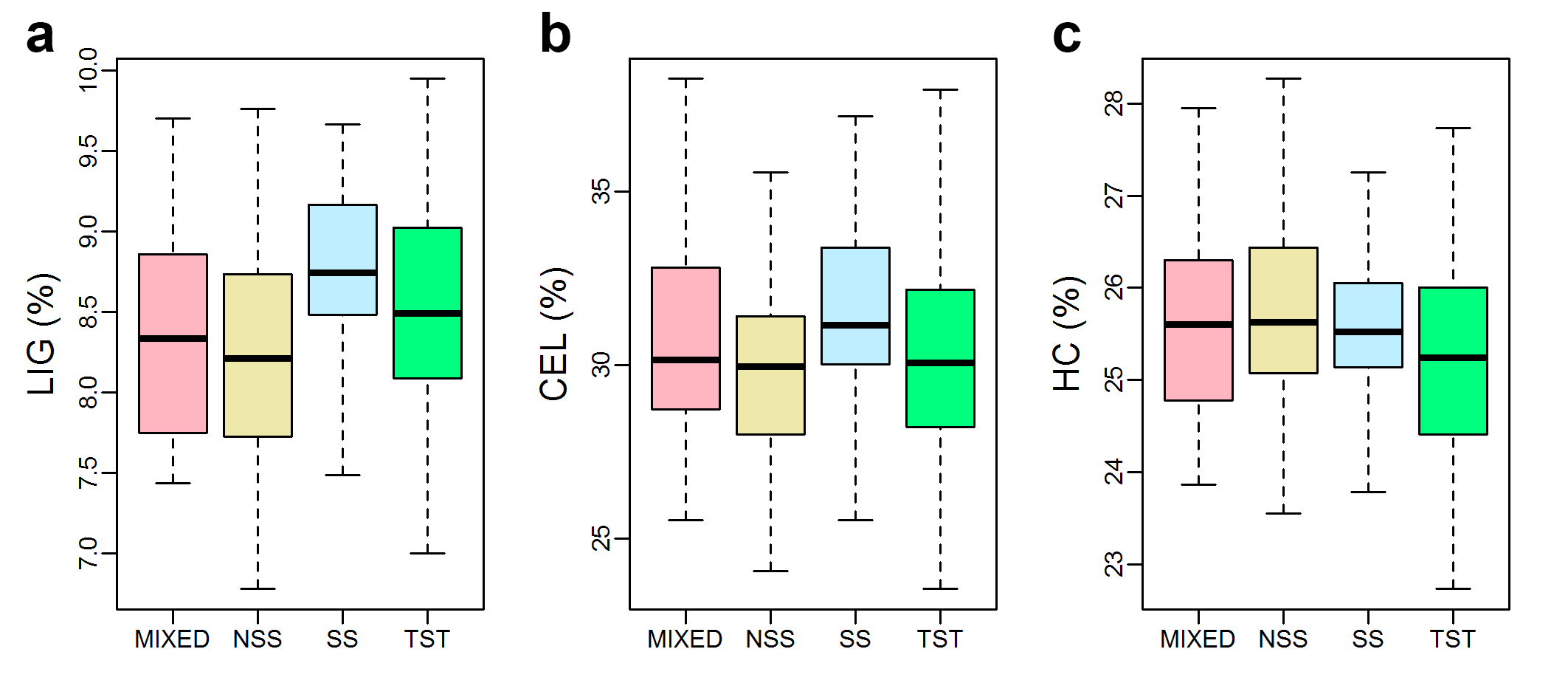

Supplement: S1 Fig — Boxplot for LIG, CEL and HC in each group are shown in a, b and c, respectively. TST, tropical and sub-tropical group; SS, stiff stalk group; NSS, non-stiff stalk group; MIXED, mixed group. (TIFF) [file pone.0158906.s001.tiff]

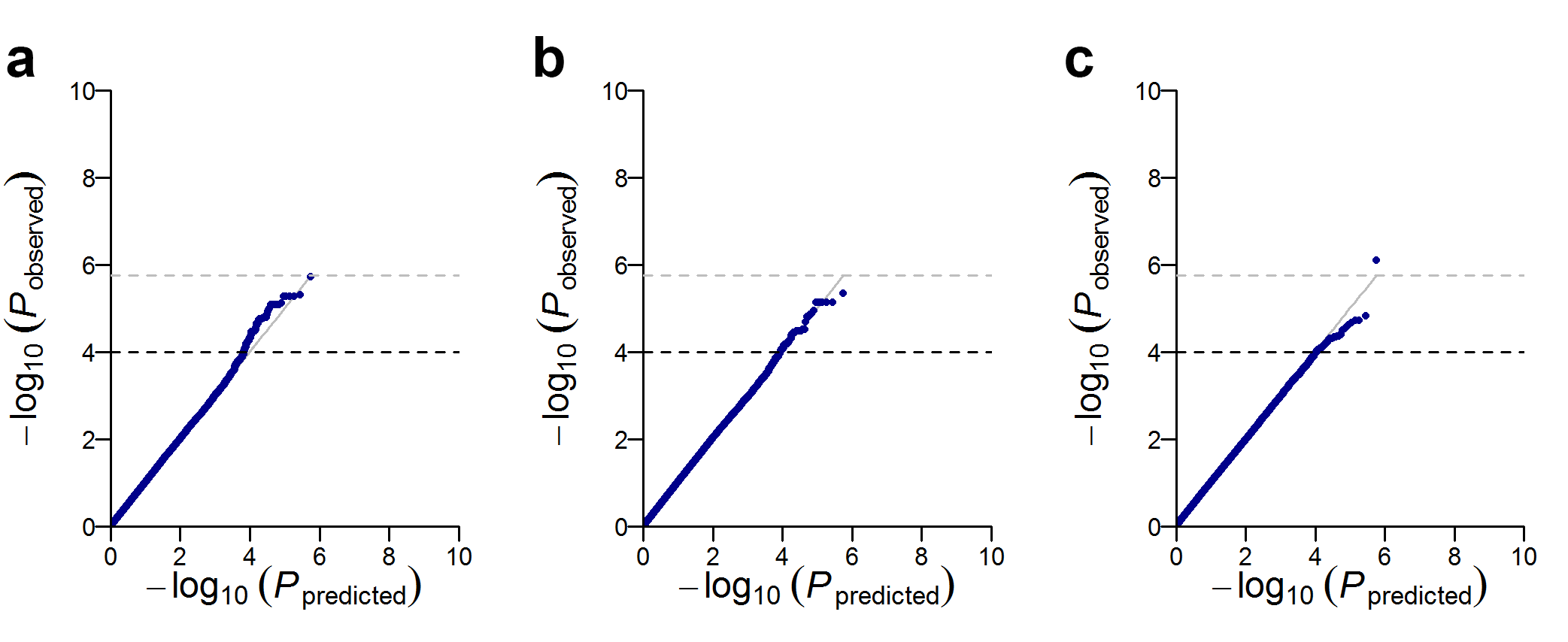

Supplement: S2 Fig — QQ plots for LIG, CEL and HC are shown in a, b and c, respectively. Horizontal grey solid line and black dashed line correspond to the thresholds of Bonferroni correction and 1×10^−4^. (TIFF) [file pone.0158906.s002.tiff]
